# Fabrication of High Thermal Conductivity Aluminum Nitride Ceramics via Digital Light Processing 3D Printing

**DOI:** 10.3390/ma17092010

**Published:** 2024-04-25

**Authors:** Yuxin Tang, Zhenhai Xue, Guohong Zhou, Song Hu

**Affiliations:** 1 State Key Laboratory of High Performance Ceramics and Superfine Microstructure, Shanghai Institute of Ceramics, Chinese Academy of Sciences, Shanghai 201899, China; 2 Center of Materials Science and Optoelectronics Engineering, University of Chinese Academy of Sciences, Beijing 100049, China

**Keywords:** DLP 3D printing, AlN ceramic, gas pressure-assisted sintering, thermal conductivity

## Abstract

The sintering of high-performance ceramics with complex shapes at low temperatures has a significant impact on the future application of ceramics. A joint process of digital light processing (DLP) 3D printing technology and a nitrogen-gas pressure-assisted sintering method were proposed to fabricate AlN ceramics in the present work. Printing parameters, including exposure energy and time, were optimized for the shaping of green bodies. The effects of sintering temperature, as well as nitrogen pressure, on the microstructure, density, and thermal conductivity of AlN ceramics were systematically discussed. A high thermal conductivity of 168 W·m^−1^·K^−1^ was achieved by sintering and holding at a significantly reduced temperature of 1720 °C with the assistance of a 0.6 MPa nitrogen-gas pressure. Further, a large-sized AlN ceramic plate and a heat sink with an internal mini-channel structure were designed and successfully fabricated by using the optimized printing and sintering parameters proposed in this study. The heat transfer performance of the ceramic heat sink was evaluated by infrared thermal imaging, showing excellent cooling abilities, which provides new opportunities for the development of ceramic heat dissipation modules with complex geometries and superior thermal management properties.

## 1. Introduction

Aluminum nitride (AlN) ceramics are attractive for their excellent properties, such as high thermal conductivity, high resistivity, and low dielectric constant. In addition, they are endowed with a thermal expansion coefficient matching that of Si [1,2,3,4]. Therefore, AlN ceramics have become one of the most popular thermal management materials in various fields, including the optoelectronic and semiconductor industries [5,6]. Conventional ceramic-shaping methods, such as dry pressing, isostatic pressing, slip-casting, tape-casting, and gel-casting, have been widely used to fabricate AlN ceramics for decades. However, these manufacturing techniques have limitations when it comes to manufacturing much more complex-shaped parts. Additive manufacturing (AM) technologies have attracted increasing scientific interest in recent years for their high degree of freedom in constructing complex shapes and highly integrated structures, which are expected to provide strong support for the design and preparation of complex-shaped ceramic parts [7,8,9,10]. Digital light processing (DLP)-based photopolymerization additive manufacturing technology has attracted much more attention to ceramic materials because it offers high manufacturing precision and surface quality. Regarding wet molding processing, property control of ceramic slurries is recognized as one of the most significant issues. A suitable liquid medium for dispersing AlN particles should be developed first. From the perspective of environmental protection, aqueous-dispersed AlN slurries have been paid special attention. However, on the one hand, the hydrolysis of AlN powders in aqueous media always passively affects the final properties of ceramics. Researchers have made efforts to shield the AlN particles from hydrolysis and achieve favorable dispersing properties, generally by blending appropriate quantities of surface-active agents, such as phosphate-based and/or carboxylic anionic surfactants [11,12]. On the other hand, water in the slurries would inevitably volatilize continuously during the long-time construction of green bodies, disturbing the stability of the slurry. Hence, as compared to the aqueous-based ceramic slurries, organic-based systems are presently more mature for the preparation of high-performance AlN ceramics via wet molding processing, especially the DLP-based additive manufacturing method.

DLP-based additive manufacturing has been extensively applied to fabricate oxide ceramics [13,14]. Nevertheless, it remains challenging when it comes to nitride ceramics, such as AlN ceramics, mainly due to the apparently higher refractive index and light-absorption properties of AlN powders. So far, there have been only a few reports on DLP 3D-printed AlN ceramics. Sheng et al. [15] integrated powder coating strategy (PCS) with DLP-based vat photopolymerization (VPP) technology to optimize the rheological and photo-curing behavior of AlN ceramic suspensions. The thermal conductivity of as-prepared AlN specimens reached 144.11 W·m^−1^·K^−1^. Lin et al. [16] selected three types of silane coupling agents as modifiers to study the effect on the rheology, stability, and curing behavior of slurries. The ceramic with a thermal conductivity of 150 W·m^−1^·K^−1^ and complex-shaped products (8~13 mm) were finally prepared. Although the researchers have made fundamental explorations, these complex-shaped ceramic products are small in size and have low thermal conductivity.

Despite the hydration-resistant treatment, the improvement in thermal conductivity of AlN ceramics is also closely related to sintering aids and sintering processes, including temperatures, holding times, and external pressures [17]. Olhero et al. [18] introduced CaF_2_/YF_3_ as a sintering adds, relatively high sintered densities (more than 96% of the theoretical density) were achieved at low sintering temperature (1750 °C) and with a short holding time (2 h). The interaction between the remaining oxygen and the sintering additives generated secondary phases, such as yttrium-rich (Y_4_Al_2_O_9_) phases, primarily located at the triple points, enhancing the thermal conductivity. The sintering of AlN ceramics mainly includes methods of pressureless sintering, hot pressing sintering, spark plasma sintering (SPS), and microwave sintering. Virkar et al. [19] used yttrium oxide as a sintering aid and obtained AlN ceramics with a relative density of 99.1% and a thermal conductivity of 194 W·m^−1^·K^−1^ after sintering at 1850 °C via pressureless sintering. Jackson et al. [20] used lanthanide oxides as sintering aids and found that the thermal conductivity of ceramics sintered at 1850 °C reached 184 W·m^−1^·K^−1^. Additionally, as the holding time was extended to 1000 min, the thermal conductivity of AlN ceramics increased to more than 200 W·m^−1^·K^−1^. Xu et al. [21] fabricated AlN ceramics with thermal conductivity exceeding 200 W·m^−1^·K^−1^ by microwave sintering at 1900 °C. Although ceramics with higher thermal conductivity can be obtained by sintering at higher temperatures or prolonging the holding time at ultrahigh temperatures, such sintering technology brings challenges for apparatuses and energy consumption control. Moreover, grain coarsening happens more easily, leading to poor mechanical properties. The process of obtaining high-performance AlN ceramics by sintering at a relatively low temperature with a shorter holding time has always been a critical issue. Kobayashi et al. [22] obtained dense AlN ceramics by using the spark plasma sintering (SPS) method; the sintering temperature was as low as 1550~1650 °C, a constant uniaxial pressure of 50 MPa was applied during the SPS process. Similarly, Feng et al. [23] used hot pressing sintering to prepare AlN ceramics with high thermal conductivity (171 W·m^−1^·K^−1^) at a low sintering temperature (1750 °C), an axial pressure of 15 MPa was also needed. However, an axial pressure is not suitable for 3D-printed samples with complex shapes. In addition, it is well acknowledged that the high density of a degreased green body is beneficial for the diffusion of grain boundaries and the exclusion of intergranular porosities. Nevertheless, for the DLP-based additive manufacturing technology, the solid loading is usually relatively low for the purpose of keeping an appropriate viscosity, resulting in a much lower density of the degreased green body, which brings greater challenges for the densification and thermal conductivity improvement.

A gas pressure-assisted sintering method was developed and verified to be one of the highly efficient strategies for the densification of nitride ceramics. An example was found in fabricating Si_3_N_4_ ceramics that with the assistance of 2~10 MPa gas pressure, the decomposition was restrained, and the diffusion of Si and N elements was promoted [24]. However, the high gas pressure has brought challenges for the sintering apparatus and cost control. In this study, AlN green bodies were prepared using the DLP 3D printing method. In order to obtain ceramics with high density and thermal conductivity at a lower sintering temperature, the gas pressure-assisted sintering method (under a lower nitrogen-gas pressure of <1 MPa) was developed to promote the densification of the DLP 3D-printed AlN ceramic bodies. The influences of the sintering temperature and gas pressure on the microstructure evolution and thermal conductivity of AlN ceramics were thoroughly discussed.

## 2. Materials and Methods

### 2.1. Materials Preparation

AlN powders (99.8%, Tokuyama Corporation, Tokyo, Japan) were the starting materials, and Y_2_O_3_ powders (99.99%, Jiangyin Jiahua Advanced Material Resources Co., Ltd., Jiangyin, China) were introduced as sintering aids. Radically photocurable monomers (Shanghai Curease Chemical Co., Ltd., Shanghai, China), including trifunctional ethoxylated trimethylolpropane triacrylate (ETPTA), ethoxylated pentaerythritol tetraacrylate (PPTTA), and 1,6-hexanediol diacrylate (HDDA), with a weight ratio of 4:1:5, were chosen as the photocurable resins, according to the former work in our research group [25]. A small amount of nonreactive polyethylene glycol (PEG) was involved as a plasticizer. Diphenyl (2,4,6-trimethylbenzoyl) phosphine oxide (TPO) was selected as the photoinitiator. AlN and Y_2_O_3_ powders were homogeneously dispersed in the photosensitive resins by planetary ball milling for 12 h, in which the weight of Y_2_O_3_ powders accounted for 4%, according to the former work in our research group [26]. AlN ceramic slurries suitable for DLP 3D printing were finally obtained after adding the photoinitiator and commercial defoamers and further ball milling for 1 h.

**Figure 1:**
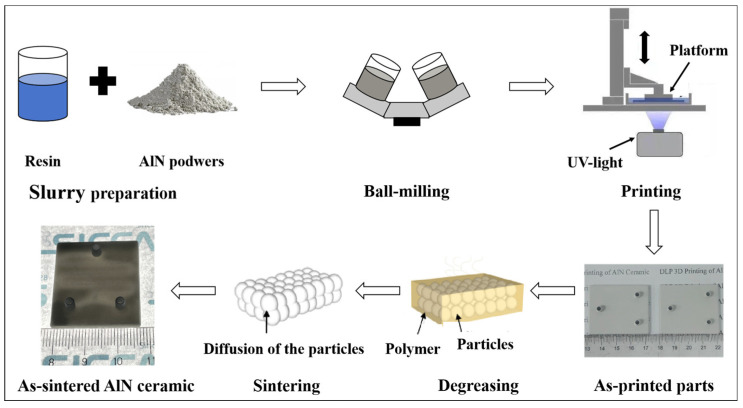
Schematic diagram of the experimental procedure for DLP 3D printing of AlN ceramic parts.

Figure 1 shows the diagram of the experimental procedure for DLP 3D printing of AlN ceramic parts. Ceramic slurries were spread out on a release film, with a layer thickness of about 100 μm, using a scraper blade. As exposed using projected UV light, the green bodies (circular sheets sized ϕ 19 mm × 2.6 mm and other complex-shaped parts) were fabricated layer by layer with a layer thickness of 20 μm based on the computer-aided designed model, using an ADMAFLEX 130 instrument (ADMATEC Europe BV, Goirle, The Netherlands), during the printing process the room temperature and humidity were kept around 24 °C and 25 RH%, respectively. The appearance of the green bodies can be found in Figure 1. The as-cleaned green bodies were heat-treated at a rate of 0.3 °C/min from room temperature to 580 °C and then held at 580 °C for 2 h in a muffle furnace to totally eliminate organic components to obtain degreased AlN samples. Part of the degreased samples were further densified by sintering at 1800 °C in a pressureless sintering condition (the obtained ceramics are abbreviated as AlN-0-1800). Some of the rest of the samples were sintered at different temperatures (1670, 1720, and 1770 °C) in a 0.2 MPa pressure-assisted nitrogen atmosphere (the obtained ceramics are abbreviated as AlN-0.2-1670, AlN-0.2-1720, and AlN-0.2-1770, respectively) and the others were sintered at 1720 °C in gas pressure assisted nitrogen atmosphere, wherein nitrogen pressures of 0.4, 0.6 and 0.8 MPa were conducted, respectively (the obtained ceramics are abbreviated as AlN-0.4-1720, AlN-0.6-1720 and AlN-0.8-1720, respectively). The specimens were double-surface polished to 1 mm for thermal diffusivity characterizations.

### 2.2. Characterization

A stress-controlled rotational rheometer (MCR302, Anton Par GmbH, Graz, Austria) with a cone (the diameter is 20 mm) was used to characterize the rheological properties of the photocurable AlN suspensions. Curing depth was determined by collecting the thickness of three independent printed samples. X-ray diffraction (XRD) measurements were performed in reflection mode on a Rigaku D/max 2500 powder X-ray diffractometer (Tokyo, Japan). The microstructures of green bodies, degreased samples, and sintered AlN ceramics were examined using fracture surfaces by scanning electron microscopy (SEM; Magellan 400, FEI, Columbia, MD, USA). Sintered AlN samples were tested using a laser thermal conductivity analyzer (LFA467 HyperFlash, Netzsch, Germany) to determine their thermal diffusivity and thermal conductivity at room temperature. The density of AlN ceramics was measured using the Archimedes method.

## 3. Results and Discussion

**Figure 2:**
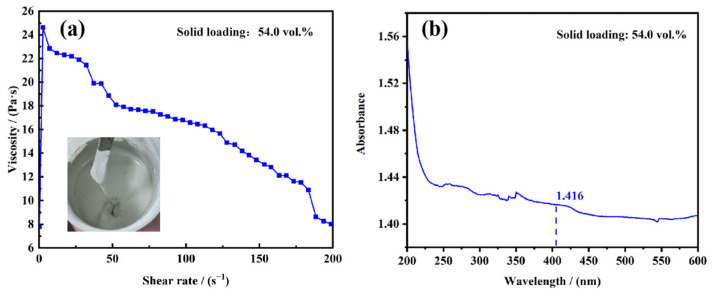
(**a**) Viscosity and (**b**) light absorptance curve of the AlN slurry with a solid loading of 54.0 vol.%, the inset of (**a**) shows the photograph of the slurry.

The solid loading of the AlN slurry was designed as 54.0 vol.%. The rheological and absorptance properties of the slurry were tested, as shown in Figure 2. It could be found in Figure 2a that the slurry exhibited non-Newtonian fluid and satisfied the shear-thinning behavior. The viscosity at 100 s^−1^ was about 16.8 Pa·s, which was suitable for spreading out uniformly but not flowing around on the foil with the scraper’s assistance. The curve in Figure 2b illustrated that the light absorbance of the slurry was tested to be 1.416 at 405 nm, which was due to the high light absorptance of AlN particles and the large difference in refractive indexes between AlN and resin monomers.

In order to explore the optimum DLP 3D printing parameters for the slurry, square slices sized 10 mm × 10 mm were cured, and the photo-curing performances were collected and evaluated, as listed in Table 1. The results showed that as the exposure time was set as 3 s, the curing depths of all groups, determined by collecting the thicknesses of three independent slices, were found quite low, even as the exposure energy increased from 40 to 60 mW·cm^−2^. The mechanical property was so poor that the photo-cured slices could not be separated from the release film. As the exposure time increased up to 6 s, both the curing depth and the mechanical property improved, and the slices could be successfully separated from the release film. However, mainly due to the high refractive index and light absorptance of the slurry, the maximum curing depth could not be higher than 40 μm by simultaneously strengthening the exposure energy and time. It is worth noting that the mechanical property could be discernibly improved. Specifically, the slice cured using the parameters of 60 mW·cm^−2^ and 12 s showed bendable characteristics. However, as the exposure energy and time were further prolonged, the curing accuracy decreased to a certain extent. This might be due to the fact that increased parameters of exposure energy and time may lead to excessive polymerization of the photosensitive resin, forming unnecessary cross-linked structures, and high exposure energy may cause light to reflect and scatter in the material, resulting in an unstable transmission path of light during the printing process, reducing the accuracy of curing. Considering the comprehensive performance of 3D printed samples, the exposure energy and exposure time were determined to be 60 mW·cm^−2^ and 12 s, respectively.

**Figure 3:**
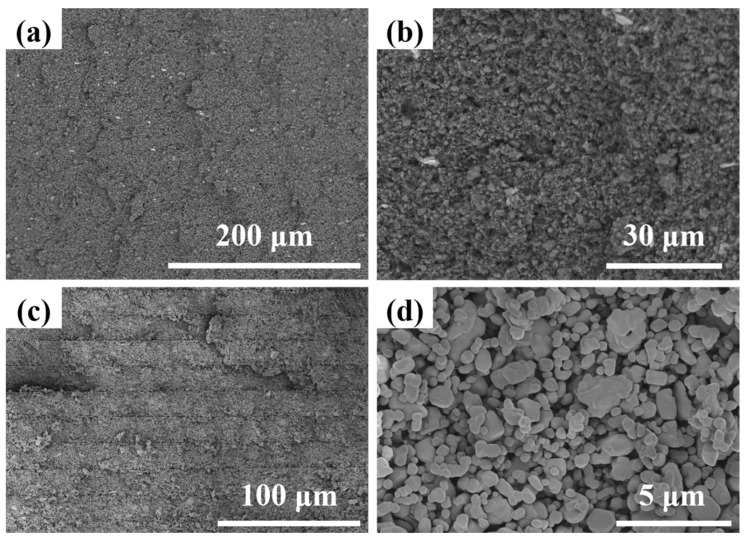
SEM images of the fracture surfaces of (**a**,**b**) AlN green bodies and (**c**,**d**) degreased AlN samples, (**b**) and (**d**) were the enlarged versions of (**a**) and (**b**), respectively.

The AlN green bodies were printed using the optimized print parameters, and the SEM images of the fracture surfaces of both as-printed AlN green bodies and degreased samples are shown in Figure 3a,b. According to Figure 3a, it could be observed that the green body presented a smooth fracture surface, much better than the samples prepared by Ozóg et al. [27], from which one could observe some layer delamination. The traces of layer-by-layer interfaces were difficult to find in Figure 3a, which illustrated that the composition of the photosensitive resin and the curing parameters were well designed. From an enlarged version in Figure 3b, even tiny defects such as micro-cracks could be found, indicating that polymerization of the ceramic suspensions was sufficient during light curing. After being degreased, it could be easily found from the fracture surface shown in Figure 3c,d that although there were traces of layer-by-layer interfaces, profiting from the excellent property of the green bodies and the elaborately designed degreasing procedure, almost no defects such as separation between layers and cracks penetrating layers could be observed even after the sample preparation procedure of breaking for observation, which was the basis for obtaining compact and defect-controlled ceramics. On the other hand, it should be noted in Figure 3d that the particles were relatively loosely packed due to the removal of the large volume ratio of organic components. Therefore, the AlN ceramics were densified.

**Figure 4:**
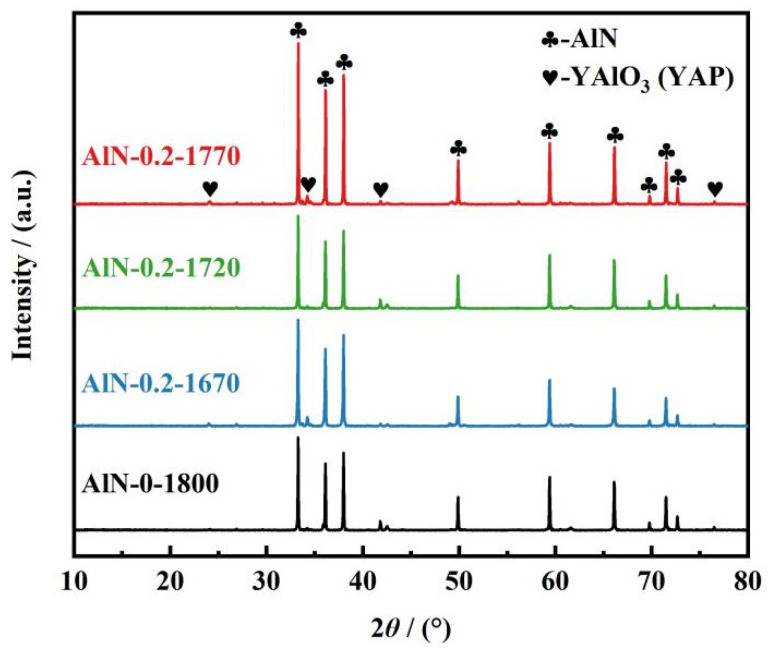
XRD patterns of AlN ceramic samples prepared using pressureless and micro-pressure assisted sintering methods.

In the present work, a nitrogen-gas pressure-assisted sintering method (under a lower nitrogen-gas pressure of <1 MPa) was proposed to promote the densification of the 3D-printed AlN ceramic parts. In order to determine the optimal sintering temperature, the degreased samples were subsequently densified via sintering at different temperatures of 1670, 1720, and 1770 °C for 4 h with the assistance of a nitrogen pressure of 0.2 MPa. For comparison, a traditional pressure-less sintering method was also conducted at 1800 °C in a flowing nitrogen atmosphere to fabricate dense AlN ceramics. XRD patterns of all the samples sintered in different conditions are presented in Figure 4. Diffraction peaks representing AlN and YAlO_3_ (YAP) phases were found in all of the AlN ceramics fabricated using different sintering methods; the Y_2_O_3_ phase was not detected, indicating that the added sintering agent Y_2_O_3_ participated in the reaction with Al_2_O_3_ to form YAP, thus the densification of AlN ceramics was promoted, and the lattice oxygen was partially eliminated [28]. The elimination of lattice oxygen was recognized to be of significant importance to the enhancement of the thermal conductivity of AlN ceramics [29].

**Figure 5:**
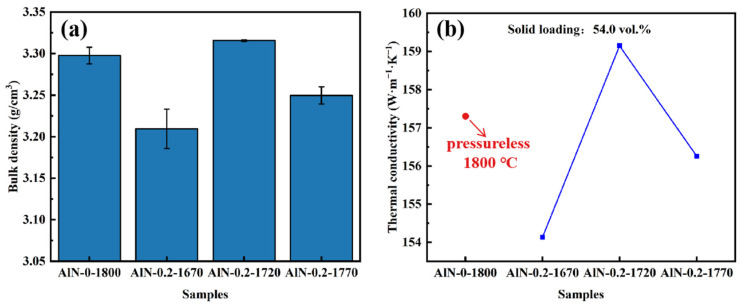
(**a**) bulk density and (**b**) thermal conductivity of AlN ceramic samples: AlN-0-1800, AlN-0.2-1670, AlN-0.2-1720, and AlN-0.2-1770.

All ceramics’ densities were measured using the Archimedes method, as displayed in Figure 5a. It could be found that for the samples sintered in a pressure-less condition at 1800 °C, the density was measured to be 3.30 g/cm^3^. For the samples sintered in a 0.2 MPa nitrogen-gas pressure-assisted condition, the density of the ceramics increased first with increasing temperature and reached a maximum of 3.32 g/cm^3^ as the temperature rose to 1720 °C, which was even higher than the ceramics sintered at 1800 °C. The thermal conductivity of the AlN ceramics also exhibits the highest value (159 W·m^−1^·K^−1^) at 1720 °C, which is relatively higher than that of the pressure-less-sintered one (157 W·m^−1^·K^−1^), as shown in Figure 5b. This proved that in spite of the ultra-high sintering temperature, the assisted nitrogen pressure was also beneficial in promoting the migration of grain boundaries and removal of residual pores, leading to higher density and thermal conductivity. The reasons accounting for differences in density and thermal conductivity between AlN-0-1800 and AlN-0.2-1720 should be traced back to the microstructures of the as-prepared ceramics.

**Figure 6:**
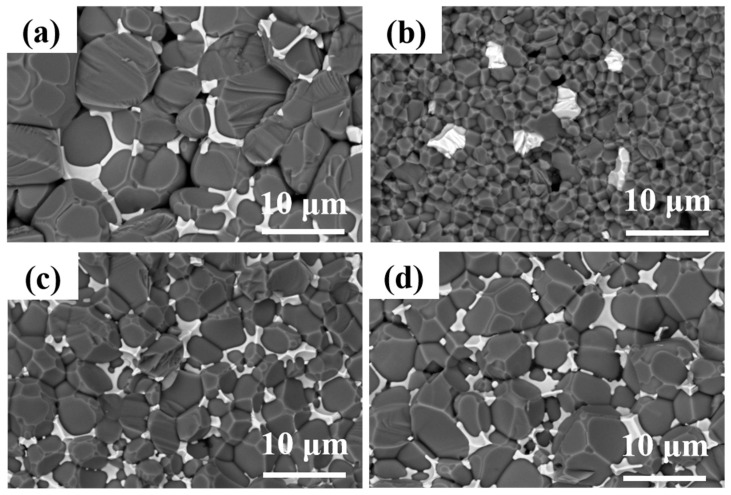
SEM micrographs of AlN ceramic samples: (**a**) AlN-0-1800, (**b**) AlN-0.2-1670, (**c**) AlN-0.2-1720, (**d**) AlN-0.2-1770.

Figure 6 shows the SEM images of fracture surfaces of the AlN ceramics. Figure 6a presents the microstructure of the sample sintered in a pressure-less condition; intergranular pores could be observed. In addition, the grain sizes were about 8 μm, larger than the ceramic samples fabricated using the micro-pressure-assisted sintering method, wherein the grain sizes were estimated to be 2, 4, and 5 μm for the samples of AlN-0.2-1670, AlN-0.2-1720, and AlN-0.2-1770, respectively, as shown in Figure 6b–d. The reason might be that a higher sintering temperature contributed to the fast grain growth in a pressure-less condition. Another obvious phenomenon was that quite a number of pores were distributed in sample AlN-0.2-1670, as shown in Figure 6b. This might be due to the fact that the low sintering temperature could not contribute to the migration of grain boundaries with a gas pressure of 0.2 MPa. Completely developed crystal grains and compact microstructures are recognized as important factors for high thermal conductivity. Furthermore, it is also well recognized that characteristics, mainly components and their distributions of grain boundaries, are another significant factor influencing thermal conductivity. It could be observed in Figure 6 that the YAP phases were distributed along the grain boundaries or gathered at the trivalent grain boundaries of AlN, except for the sample of AlN-0.2-1670; only traces of secondary phases were observed among the AlN ceramic grains, which might be attributed to the relatively low sintering temperature. As the sintering temperature increased to 1720 °C, residual pores were overwhelmingly removed; as shown in Figure 6c, the secondary phase of YAP was distributed mainly at the trivalent grain boundaries, leading to improved density and heat conduction properties of the ceramic. As the sintering temperature rose to 1770 °C, a higher proportion of the secondary phase was distributed along the AlN grains rather than gathering at the trivalent grain boundaries. Moreover, more intergranular pores could be observed. This might be due to the fact that, with the assistance of a micro-pressure atmosphere, an increase in temperature by 50 °C contributed to the fast grain growth as compared to that of the AlN-0.2-1720 ceramic, resulting in abnormal grain growth and residual pores, which also accounted for the decrease in both density and thermal conductivity. According to the above phenomena, 1720 °C was chosen as the optimal sintering temperature for further study.

**Figure 7:**
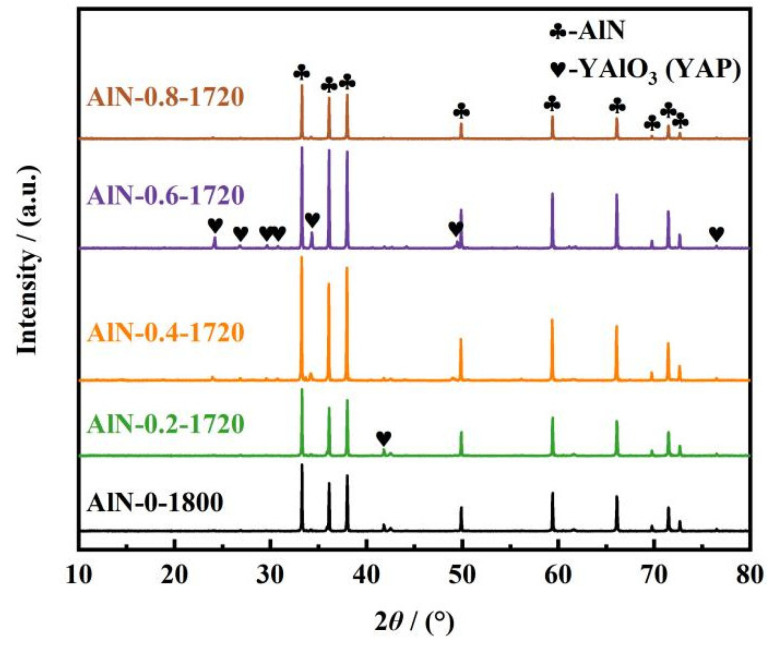
XRD patterns of AlN ceramic samples: AlN-0-1800, AlN-0.2-1720, AlN-0.4-1720, AlN-0.6-1720, and AlN-0.8-1720.

To investigate the effect of nitrogen pressure on the densification of AlN ceramics, in the case that the sintering temperature was determined to be 1720 °C, another three groups of degreased samples were sintered with the assistance of 0.4, 0.6, and 0.8 MPa nitrogen pressure, respectively. It could be observed in Figure 7 that all AlN ceramic samples were composed of AlN and YAP phases. The relative intensity of the crystalline peaks of YAP was the highest for the AlN-0.6-1720 ceramic. However, the XRD results of the sample sintered at 0.8 MPa showed faint crystalline peaks of YAP.

**Figure 8:**
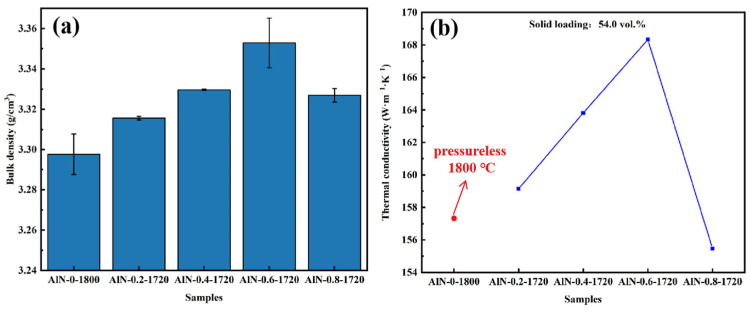
(**a**) bulk density and (**b**) thermal conductivity of AlN ceramic samples: AlN-0-1800, AlN-0.2-1720, AlN-0.4-1720, AlN-0.6-1720, and AlN-0.8-1720.

The density shown in Figure 8a displayed a tendency to rise and then fall with the increase in pressure; the maximum was 3.35 g/cm^3^ for the AlN-0.6-1720 ceramic. According to Figure 8b, the curve of the ceramics’ thermal conductivity indicated the same trend as that of density, which reached the maximum of 168 W·m^−1^·K^−1^ for the AlN-0.6-1720 sample. In addition, the densities and thermal conductivities of all micro-pressure-assisted sintered samples were higher than those of the pressure-less-sintered ones, which proved that the gas pressure-assisted sintering method could effectively realize the fabrication of high-performance ceramics at a suitable temperature. However, the thermal conductivity of the AlN-0.8-1720 sample decreased sharply, which was even lower than that of the pressure-less-sintered ones. The phenomenon could be further explained by examining the microstructures of as-prepared ceramics.

**Figure 9:**
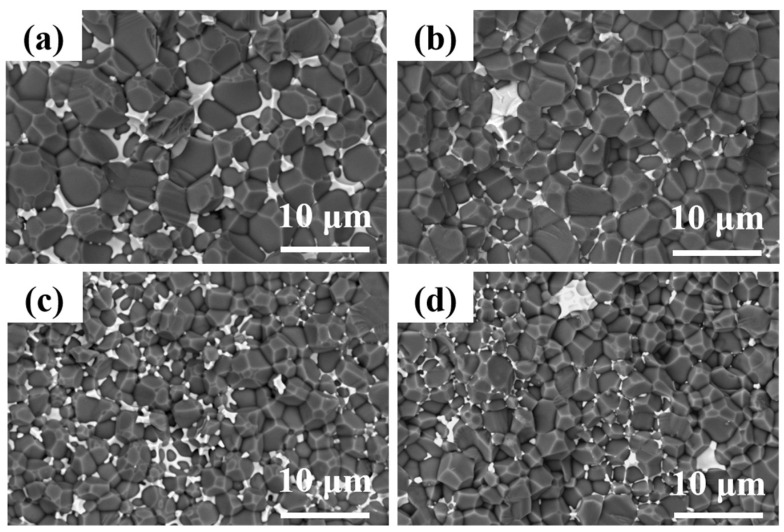
SEM micrographs of AlN ceramic samples: (**a**) AlN-0.2-1720, (**b**) AlN-0.4-1720, (**c**) AlN-0.6-1720, (**d**) AlN-0.8-1720.

The microstructure shown in Figure 9 further illustrates the role of the gas pressure-assisted sintering method. With the increase in nitrogen pressure, the AlN grain size became much smaller. Specifically, as the pressure increased, the secondary phases were found to be more inclined to gather at the trivalent grain boundaries. As the secondary phase is continuously distributed along the grain boundaries, the AlN grains are separated, and the phonon scattering increases, leading to a decline in thermal conductivity. On the contrary, as the secondary phase is gathered at the trivalent grain boundaries, more AlN grains can be in direct contact with each other, leading to higher thermal conductivity. Therefore, the AlN-0.6-1720 ceramic exhibited the highest thermal conductivity. Nevertheless, the appearance of the YAP phase decreased obviously as the nitrogen pressure rose to 0.8 MPa, as shown in Figure 9d. This might be due to the fact that with the increase in nitrogen pressure, the diffusion ability of the secondary phase was enhanced, which may have led to the fast accumulation of YAP at the surface of the ceramic sample before it actually played a role in promoting the densification of AlN ceramics. Therefore, both the density and thermal conductivity of the ceramics sintered at 1720 °C with the assistance of a nitrogen pressure of 0.8 MPa decreased as compared to the AlN-0.6-1720 sample.

**Figure 10:**
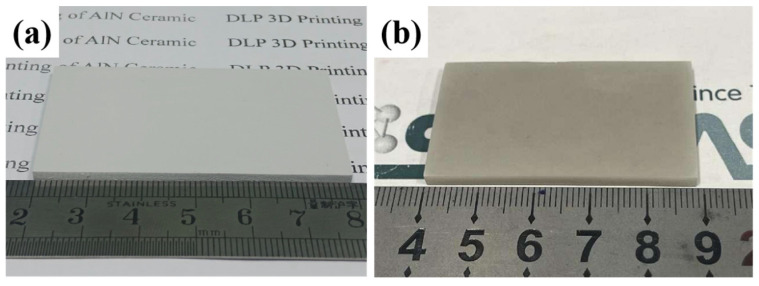
(**a**) Green body, (**b**) sintered sample of 3D printed large-sized AlN components.

In order to demonstrate the possibilities opened up by a joint process of DLP 3D printing and the gas pressure-assisted sintering method, the sintering temperature and gas pressure were determined to be 1720 °C and 0.6 MPa, respectively, large-sized and complex-shaped AlN ceramic parts were further fabricated. Figure 10a shows the appearance of a 3D printed AlN green body with a size of 59 mm × 34 mm × 4 mm; the as-sintered sample shown in Figure 10b has a size of about 49 mm × 28 mm × 3 mm. As can be observed from the images, the surfaces of the green bodies and sintered components were uniform without defects such as cracking, transformation, and large amounts of porosity, demonstrating that the proposed gas pressure-assisted sintering method could be applied to fabricate 3D-printed large-sized AlN ceramic parts.

**Figure 11:**
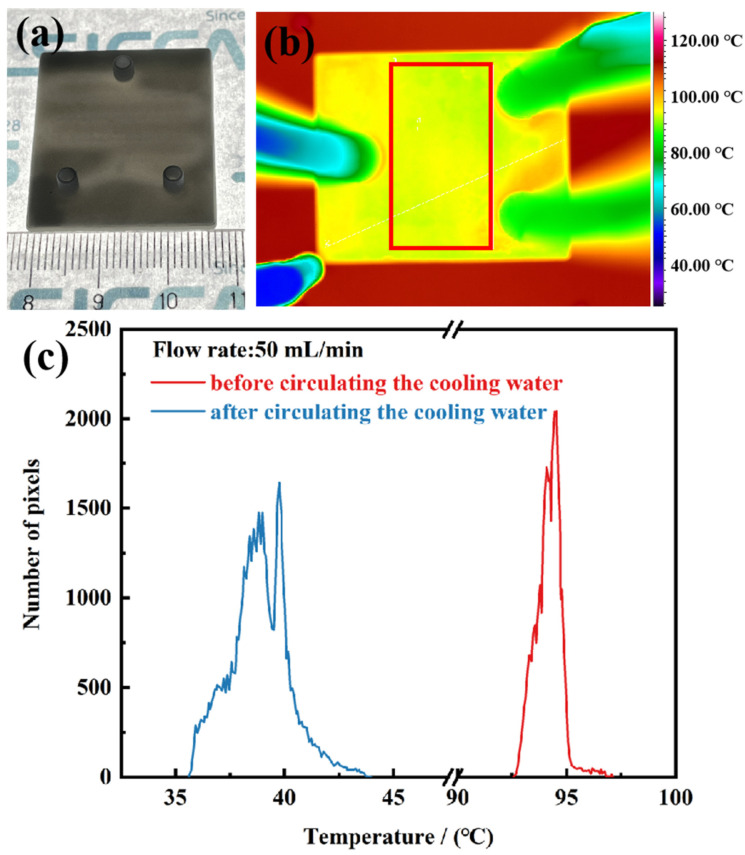
(**a**) The 3D printed AlN ceramic heat sink with an internal mini-channel structure, (**b**) infrared thermal imaging of the ceramic heat sink with flowing water attached to the heating stage at 110 °C, and (**c**) temperature distribution in the rectangular area before and after circulating a certain flux of 50 mL/min cooling water.

AlN ceramic heat sinks with an internal mini-channel structure were further designed and successfully fabricated using optimized printing and sintering parameters. The physical photographs of sintered ceramic are shown in Figure 11a. Further insight from the thermal imaging figure shown in Figure 11b indicated that almost no macroscopic defects could be found across the ceramic heat sink, and once the flowing water was pumped from the inlet into the channels, the surface temperature of the ceramic heat sink dropped rapidly from higher than 95 °C to about 40 °C (recorded using the IR camera) as it was attached to the heating stage of 110 °C, indicating that the high-efficiency heat transfer of the ceramic heat sink could be achieved. Furthermore, although the mini-channels were not elaborately designed, the temperature distributions were quite narrow before and after the coolant was pumped, as illustrated in Figure 11c, indicating that the as-fabricated AlN ceramic heat sinks possessed excellent comprehensive properties, including uniform microstructures and high thermal conductivity, making them promising for the thermal management of high power devices.

## 4. Conclusions

AlN ceramics with high density and thermal conductivity were successfully fabricated using DLP 3D printing together with a micro-pressure-assisted sintering method. The optimum exposure energy and time for printing were determined to be 60 mW·cm^−2^ and 12 s, respectively. The effects of the sintering temperature and nitrogen pressure on the microstructure, density, and thermal conductivity of the AlN ceramics were thoroughly investigated. The sintered samples consist of major AlN and minor YAP phases, wherein the formation of YAP from Y_2_O_3_ and Al_2_O_3_ contributed to the densification of the AlN ceramics. The optimum sintering temperature was determined to be 1720 °C with the assistance of a low nitrogen-gas pressure less than 1 MPa. Compared with the ceramics sintered at higher temperatures in a pressure-less condition, the density and thermal conductivity of the ceramics obtained using the nitrogen gas pressure-assisted sintering method were extensively improved. This was due to the fact that the diffusion ability of the secondary phase was enhanced under gas pressure. With properly controlled sintering temperature and gas pressure, the secondary phases could tend to gather at the trivalent grain boundaries, contributing to higher thermal conductivity. The AlN ceramics with a density of 3.35 g/cm^3^ and thermal conductivity of 168 W·m^−1^·K^−1^ were finally obtained by sintering at 1720 °C with the assistance of a 0.6 MPa gas pressure nitrogen atmosphere, verifying the feasibility of micro-pressure assisted sintering method for promoting densification of nitride ceramics. Large-sized AlN ceramic plates and heat sinks with internal mini-channel structures were further successfully designed and fabricated without obvious defects. With the proper fluid flux of cooling water, the equilibrium temperature of the ceramic surface could be reduced rapidly from a high-temperature heating source, indicating that the high-efficiency heat transfer of the AlN ceramic heat sink could be achieved. In the near future, the technology developed in the present work is promising for the fabrication of high-performance thermal management materials for high-power or high-power-density opto-electronic components.

## Figures and Tables

**Table 1:** The behavior of the photosensitive AlN slurries cured with different parameters of exposure energy and exposure time (the curing depth was determined by collecting the thickness of three independent slices).

Sample	Exposure Energy (mW·cm^−2^)	Exposure Time (s)	Curing Depth (μm)	Mechanical Property
1	40	3	/	poor
2		6	40	poor
3		9	40	poor
4		12	40	moderate
5	45	3	/	poor
6		6	40	poor
7		9	40	moderate
8		12	40	moderate
9	50	3	/	poor
10		6	40	moderate
11		9	40	moderate
12		12	40	moderate
13	60	3	/	poor
14		6	40	moderate
15		9	40	moderate
16		12	40	excellent

## Data Availability

Data is contained within the article.
